# Effectiveness of point of care ultrasound (POCUS) simulation course and skills retention for Japanese nurse practitioners

**DOI:** 10.1186/s12912-023-01183-2

**Published:** 2023-01-23

**Authors:** Toru Yamada, Jun Ehara, Hiraku Funakoshi, Keita Endo, Yuka Kitano

**Affiliations:** 1 Department of General Medicine, Graduate School of Medical and Dental Sciences, Tokyo Medical and Dental University, Bunkyo-Ku, Tokyo, 113-8510 Japan; 2 Department of Internal Medicine, Tokyo Bay Urayasu Ichikawa Medical Center, Urayasu, Chiba Japan; 3 Department of Emergency and Critical Care Medicine, Tokyo Bay Urayasu Ichikawa Medical Center, Urayasu, Chiba Japan; 4 Department of Nephrology, Endocrinology and Diabetes, Tokyo Bay Urayasu Ichikawa Medical Center, Urayasu, Chiba Japan; 5 Department of Emergency and Critical Care Medicine, St. Marianna University School of Medicine, Kawasaki, Kanagawa Japan

**Keywords:** Simulation training, Action record, Behavior change, Skills retention, Point-of-care ultrasonography, Nurse practitioner

## Abstract

**Background:**

In Japan, the nurse practitioner (NP) system has only been in place for a short time, and there is no ultrasound (US) simulation course for NPs. Therefore, NPs may have to attend US simulation courses for physicians. We evaluated whether US simulation course for physicians lead to improved image acquisition and interpretation amongst NPs and, if so, if these changes would be maintained over time.

**Methods:**

A 2-day point-of-care ultrasound (POCUS) course designed for physicians in cardiac US, lung US, lower extremity deep vein thrombosis (DVT) US, and abdominal US was held for Japanese nurse practitioners (JNP) and JNP trainees in 2018 and 2019. Participants kept a record of the number of US examinations they performed for 3 months before and 3 months after the course. The number of US exams performed was grouped into six categories. All participants underwent pre-course, immediate post-course, and 4-month post-course testing to assess image interpretation skills, image acquisition skills, and confidence.

**Results:**

Thirty-three participants from 21 facilities completed the program. Before and immediately after the course, test scores of the image interpretation test, image acquisition test, and confidence increased significantly (37.1, 72.6: *P* < 0.001), (13.7, 53.6: *P* < 0.001), and (15.8, 35.7: *P* < 0.001), respectively. Comparing the follow-up tests immediately after the course and 4 months later, there was no decrease in scores on the image interpretation test, the image acquisition test, or confidence (72.6, 71.8: *P* = 1.00) (53.6, 52.9: *p* = 1.00) (35.7, 33.0: *P* = 0.34). There was a statistically significant increase (*P* < 0.001) in both the total number of ultrasound examinations and in the number of ultrasound examinations by category (cardiac, lung, lower extremity DVT, and abdominal) in the 3 months before and 3 months after the course.

**Conclusions:**

The POCUS simulation course for physicians is useful for JNPs to acquire US examination skills even if it is not arranged for JNPs. Image interpretation skill, image acquisition skill, and confidence improved significantly and were maintained even after 4 months of the course. It leads to behavioral changes such as increasing the number of US examinations in daily practice after the course.

**Supplementary Information:**

The online version contains supplementary material available at 10.1186/s12912-023-01183-2.

## Background

Point-of-care ultrasonography (POCUS) has become widespread in various fields, including emergency departments, wards, and intensive care units. POCUS is used for echo-guided procedures as well as diagnosis, and contributes to reducing complications [1, 2]. POCUS is also part of under-graduate and post-graduate medical education [3–5]. Numerous POCUS simulation courses with variable content and duration are available [6, 7]. Generally, simulation courses such as the Advanced Cardiovascular Life Support course have been shown to increase knowledge or skills [8]. POCUS simulation courses for medical students and doctors have shown similar results [1, 5, 7, 9]. Few studies have evaluated the educational effect of POCUS courses for nurse practitioners (NPs); however, some studies reported that this training improved skills [10–12]. In Japan, NP is still a young profession, started in 2015, and its number is still small. Therefore, there are very few medical simulation courses for Japanese nurse practitioners (JNP), and especially POCUS simulation courses have not existed in Japan until now. The training requirements of JNP are mainly for procedures such as intubation and arterial blood sampling, and since there are no clear requirements for knowledge, knowledge backgrounds such as anatomy and physiology may differ greatly even among JNPs. Therefore, it should be confirmed whether POCUS simulation courses for physicians are also useful for JNPs.

**Fig. 1:**
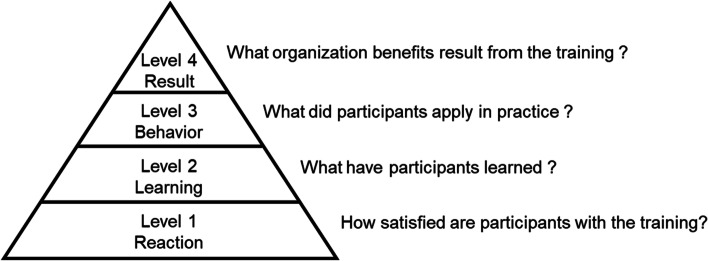
Four levels of learning evaluation. Figure created with reference to Kirkpatrick and Kirkpatrick (2007)

In evaluating the educational effect of training, the four levels of learning evaluation was advocated by Kirkpatrick. His model argues that practical application (level 3; behavior) is more important than the information learned (level 2; learning) (Fig. 1) [13]. Few studies have shown that simulation training can change both behavior and the learning level, especially regarding POCUS for NPs [14]. In addition, simulation courses can increase knowledge and skills immediately after the course; however, these gains tend to decline a few months after the training [4, 8, 9, 15, 16]. To resolve this problem, follow-up lectures or hands-on training after the initial course may be effective for maintaining knowledge [17–19]. POCUS simulation courses are similar; however it is unclear whether both knowledge and clinical skills (e.g., image acquisition) can be maintained [4, 5, 9, 15, 16, 20, 21].

In this study, we held a 2-day POCUS simulation course for JNPs and JNP trainees. These practitioners were instructed to record the number of ultrasound (US) examinations they performed before and after the course. This study had two aims. First, we evaluated whether the POCUS simulation course for physicians was useful for JNPs in terms of the three components of US examination skills: image acquisition skills, image interpretation skills, and confidence score. Second, we aimed to examine whether keeping a record of the number of US examinations would help maintain US knowledge and skills and lead to behavioral change (Level 3: Behavior) of increasing the number of US examinations in daily practice. Behavior change was evaluated by comparing the number of US exams performed before and after the course. To evaluate maintenance of the learning level, we evaluated image interpretation skills, image acquisition skills, and confidence in performing POCUS before and immediately after the course, and again 4 months after the course.

## Methods

### Study design and setting

**Fig. 2:**
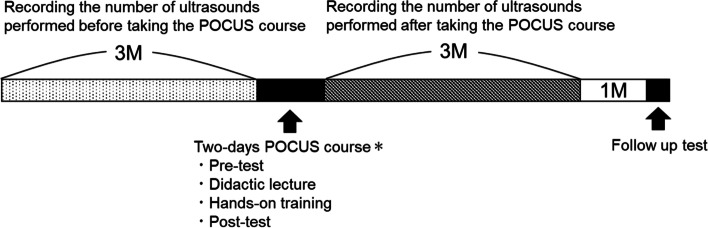
Course design *(Yamada et al. 2018)

We held one POCUS training program in 2018 and one in 2019. The program involved four parts: 1) recording the number of US exams performed during the 3 months before participating in the POCUS course, 2) participating in the 2-day POCUS course, 3) recording the number of US exams performed during the 3 months after participating in the course, and 4) follow-up evaluation 4 months after the course. All participants recorded the number of cardiac US, lung US, deep vein thrombosis (DVT) US for the lower extremities, and abdominal US they performed in the 3 months before the course. We chose these four US examinations because the 2-day POCUS course focused on these examinations A standardized POCUS course with a proven educational effect was adopted for our 2-day POCUS course [7]. The educational effects of this course for medical students and doctors have been demonstrated; however, this was the first such training for JNPs [7]. Participants’ image interpretation skills, image acquisition skills, and confidence in performing POCUS were evaluated before and after the course. Image interpretation skills were evaluated by a written examination using POCUS case study videos and multiple-choice questions [7]. It consisted of 20 questions on the basics of ultrasound and 10 questions on case studies. For each question, the participants watched an ultrasound video and answered multiple choice questions. Image acquisition skills were evaluated by hands-on using live models. The evaluation domains were cardiac US, lung US, deep vein thrombosis (DVT) US for the lower extremities, and abdominal US. For each domain, the participant performed image acquisition and the instructor evaluated it using an evaluation sheet (Additional file 1). Confidence was evaluated by a self-evaluation sheet with a five-point Likert scale using previously validated multiple-choice questions and a self-evaluation survey (Additional file 2) [7]. Participants then recorded the number of US examinations they performed for the 3 months after the course. Four months after completing the course, participants completed a follow-up test covering image interpretation skills, image acquisition skills, and confidence. The follow-up test used the same format and number of questions as the pre- and post-tests for image interpretation skill, image acquisition skill, and confidence, but the questions for the image interpretation skill test were changed (Fig. 2). There were no interventions, including didactic lectures, between the end of the course and the 4-month follow-up test.

Nine instructors were involved in the course and evaluated participants. All instructors were certified POCUS instructors [7]. Before the course, the instructors received a lecture presenting the evaluation method and online discussions to standardize the evaluation method.

This study was approved by the Institutional Review Board of the Tokyo Bay Urayasu Ichikawa Medical Center. Before participation, participants were informed that the results of this study would not affect evaluation of their work or future training. Written informed consent was obtained from all participants.

## Participants

Japan has an original nurse practitioner system (JNP system), which began in 2008 and was partially revised in 2015. [22]. JNPs are licensed nurses who have received training and approval for specific medical procedures. There are several certified JNP training programs in Japan. In the present study, JNPs and JNP trainees were recruited through the JNP training program delivered by the Japan Association for the Development of Community Medicine from 2018 to 2019. During the study period, JNPs worked in hospitals or clinics, and JNP trainees worked in hospitals and were engaged in on-the-job training under the supervision of attending doctors; therefore, all participants could access portable US machines and perform US examinations. The type of facility (hospital or clinic) and the department to which the JNPs participating in the study belonged were not limited. All ultrasound examinations were performed under the supervision of a physician.

## Data collection

All participants recorded the number of US examinations they performed during the three months prior to attending the two-day POCUS course, categorized the number into categories, and reported the results to the Secretariat (category 1: 0 cases, category 2: 1–9 cases, category 3: 10–29 cases, category 4: 30–49 cases, category 5: 50–99 cases, and category 6: ≥ 100 cases). Image interpretation skills, image acquisition skills, and confidence in performing POCUS were evaluated pre- and immediately post-course. Participants recorded the number of US examinations they performed for 3 months after the course, using the Microsoft Excel sheet (Additional file 3) distributed by the study secretariat. These records were collected by the study secretariat. Four months after the course, participants completed follow-up testing to evaluate their image interpretation skills, image acquisition skills, and confidence.

## Statistical analysis

Comparisons of the difference between the US examination categories before and after the course were analyzed using Wilcoxon’s signed-rank test. Written examinations, evaluation sheets, and self-evaluation survey scores were analyzed with the Friedman test with Bonferroni adjustment. Data analyses were performed using EZR statistical software (version 1.52), which is a graphical user interface for R (The R Foundation for Statistical Computing, Vienna, Austria) [23].

## Results

### Participants

Thirty-five participants completed the POCUS training program in 2018 or 2019. Two participants were excluded because they could not complete the program. Nine JNPs and 24 JNP trainees from 21 facilities completed the program. These facilities were geographically distributed across Japan from Hokkaido in the north to Nagasaki prefecture in the south. Some participants were from the same facilities, and most (94%) worked in community hospitals in the remote areas; no participants worked in university hospitals. The mean number of post-graduate years was 13.2 years (range: 6–22 years). All participants were novice POCUS trainees (Table 1).

**Table 1:** Participants’ characteristics (*n* = 33)

Characteristic	Total (%)	JNP^a^ (%)	JNP trainee (%)
Number of participants	33	9	24
Sex			
Male	10 (30)	3 (33)	7 (29)
Female	23 (70)	6 (66)	17 (71)
Work environment			
Clinic	2 (6)	0 (0)	2 (8)
Community hospital	31 (94)	9 (100)	22 (92)
University hospital	0 (0)	0 (0)	0 (0)
Post-graduate year			
1–5	0 (0)	0 (0)	0 (0)
6–10	10 (30)	3 (33)	7 (29)
11–15	10 (30)	4 (45)	6 (25)
≥ 16	13 (40)	2 (22)	11 (46)
Novice POCUS^b^ trainee^c^	33 (100)	9 (100)	24 (100)

^a^*JNP* Japanese nurse practitioner

^b^*POCUS* Point-of-care ultrasound

^c^Participants who had never participated in a POCUS simulation course

## Number of US examinations performed before and after the course

**Fig. 3:**
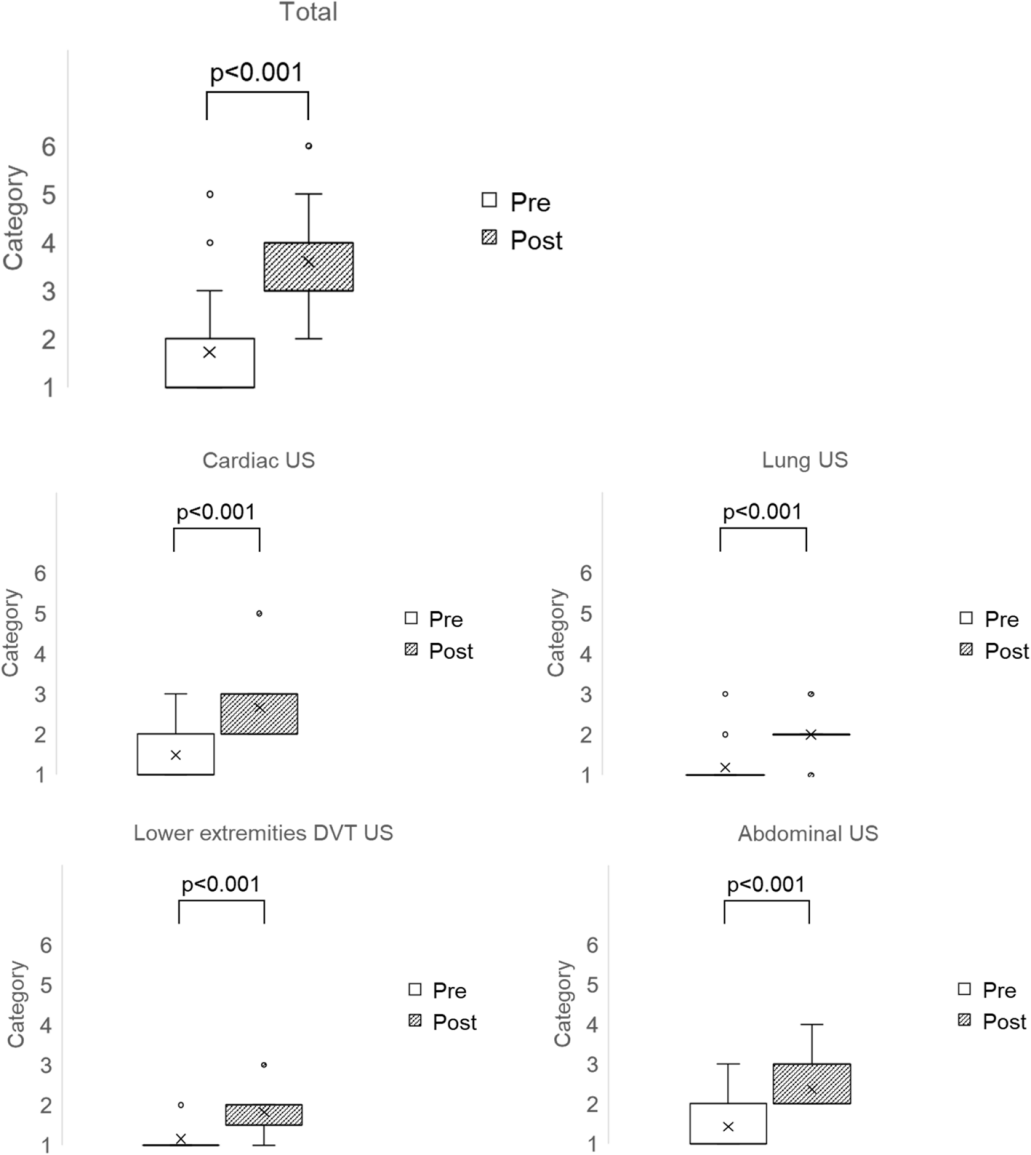
Box plot of the number of ultrasound examinations performed for 3 months before and after the course. Category 1: 0 cases, category 2: 1–9 cases, category 3: 10–29 cases, category 4: 30–49 cases, category 5: 50–99 cases, category 6: ≥ 100 cases

The median category for the number of US examinations performed 3 months before the course was category 1 (standard deviation [SD]: 1.0) and that for US performed 3 months after the course was category 3 (SD: 0.9). The median category after the course had statistically significantly increased compared with before the course (*P* < 0.001). The median categories for the 3 months before the course for cardiac US, lung US, lower extremities DVT US, and abdominal US examinations were 1 (SD: 0.7), 1 (SD: 0.5), 1 (SD: 0.4), and 1 (SD: 0.7), respectively. The median categories 3 months after the course for cardiac US, lung US, lower extremity DVT US, and abdominal US examinations were 3 (SD: 0.6), 2 (SD: 0.4), 2 (SD: 0.5), and 2 (SD: 0.6), respectively. The median category for each US examination type 3 months after the course had statistically significantly increased compared with before the course (*P* < 0.001) (Fig. 3). (Additional files 4, 5).

## Image interpretation skills, image acquisition skills, and confidence scores

**Fig. 4:**
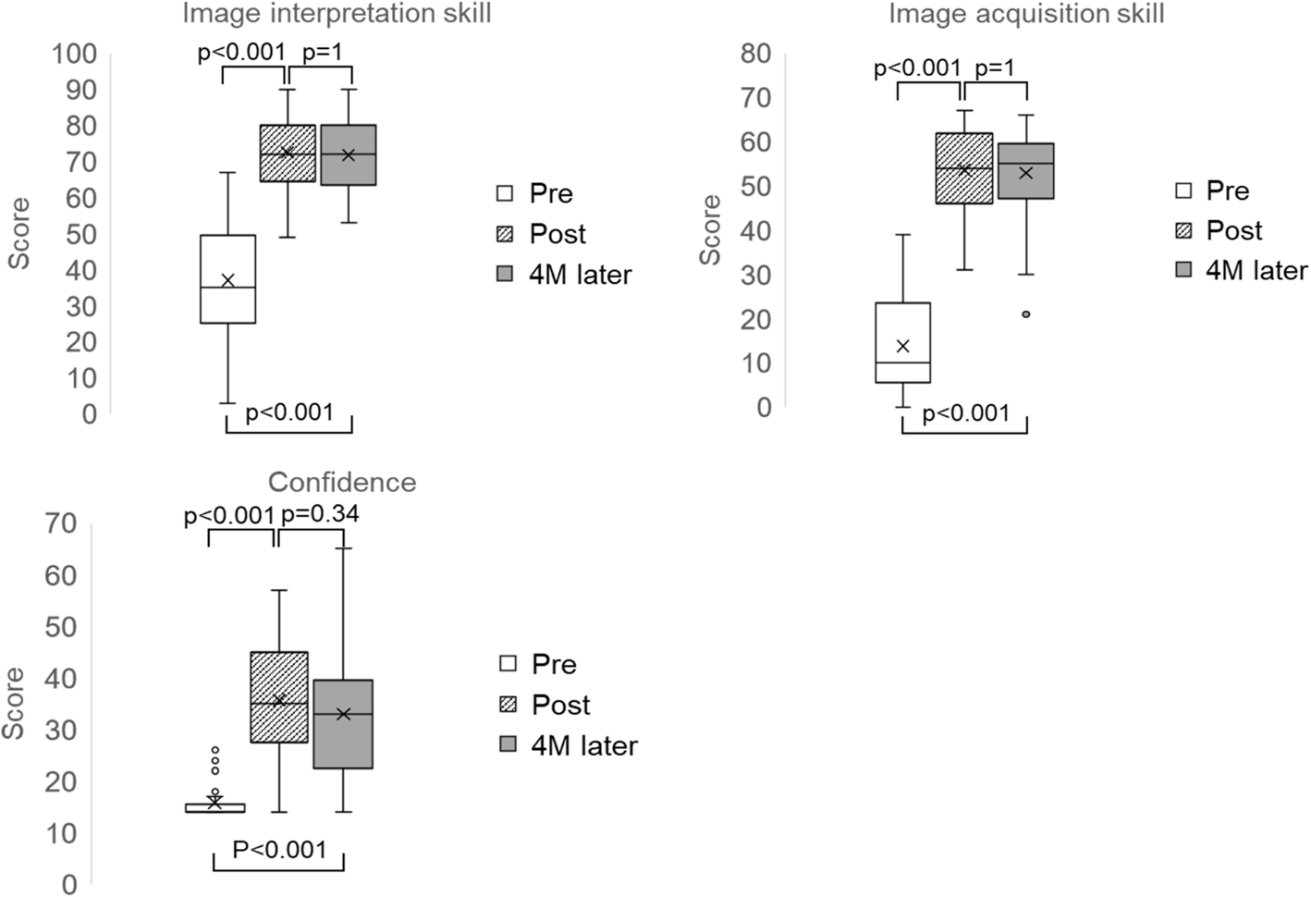
Box plot of the results of the written examination (image interpretation skills), evaluation sheets (image acquisition skills), and self-evaluation survey scores (confidence) before and immediately after the course and 4 months after the course. M; months

The mean scores for the image interpretation skills test pre-course, and immediately post-course, and at the 4-month follow-up evaluation were 37.1 (SD: 16.0), 72.6 (SD 11.1), and 71.8 (SD 9.9) (out of 100 points), respectively. Both the immediate post-course test and the 4-month follow-up test scores were statistically significantly higher than the pre-course scores (*P* < 0.001). However, the difference between the immediate post-course and the 4-month follow-up test scores was not statistically significant (*P* = 1.00). The mean scores for the image acquisition skills test pre-course, immediate post-course, and at the 4-month follow-up were 13.7 (SD: 10.7), 53.6 (SD: 8.9), and 52.9 (SD: 9.3) (out of 71 points), respectively. Both the immediate post-course and 4-month follow-up test scores were statistically significantly higher than the pre-course test scores (*P* < 0.001). The difference between the immediate post-course and the 4-month follow-up test scores was not statistically significant (*P* = 1.00). The mean scores for confidence pre-course, immediate post-course, and at the 4-month follow-up survey were 15.8 (SD: 3.6), 35.7 (SD: 10.5), and 33.0 (SD: 11.6) (out of 70 points), respectively. Both the immediate post-course survey and 4-month follow-up test scores were statistically significantly higher than the pre-course survey scores (*P* < 0.001). The difference between the immediate post-course and the 4-month follow-up test scores was not statistically significant (*P* = 0.34) (Fig. 4).

## Discussion

In this study, we evaluated whether a POCUS simulation course for physicians was effective for JNPs, the extent to which US knowledge and skills were maintained after attending the course, and whether the number of ultrasound examinations performed in routine clinical practice changed. The results showed that the POCUS course for physicians was significantly useful in improving JNPs' US skills. POCUS knowledge, skills, and confidence were maintained even 4 months after attending the course. In addition, attending the course significantly increased the number of US examinations performed in daily practice and led to behavior change.

JNP has only been in place for a short time, and the training system is not yet brushed up. However, although government shows regulations, the content of the training is left up to the training facility to some extent, and the quality of the training varies [22]. In addition, because the number of JNPs is small and the history is young, there are no simulation courses specifically for JNPs, and the current situation is that JNPs are forced to use the educational content of doctors. To what extent the educational content for physicians is useful for JNPs with a nursing license needs to be verified and clarified one by one. In Japan, it is not common for nurses to perform US examinations. However, for JNPs who are expected to be active in areas where there is a shortage of physicians, US examination skills are a very useful, and learning POCUS is a great advantage [22]. This study showed that the POCUS course for physicians is significantly useful for JNPs even if it is not arranged for JNPs. POCUS is characterized by its limited number of evaluation items for each organ and simplified evaluation methods compared to comprehensive ultrasound examinations, making it easier to learn. Past reports have shown that there was no difference in the level of mastery between residents and supervisors [7].These characteristics of POCUS may be the reason why the POCUS course for physicians was also useful for JNPs. It is hoped that this will be a major impetus for JNPs to learn POCUS.

In educational methods, including in simulation training, it is important and most effective to cause both a reaction or learning improvement (Kirkpatrick’s levels 1 and 2, respectively), and behavior change or improvement (Kirkpatrick’s levels 3 and 4, respectively) [13]. However, it is often difficult to evaluate levels 3 and 4 because this evaluation is time consuming and requires effort and cost to train evaluators and prepare tools and facilities. Therefore, few studies have evaluated behavior change, and effective methods to change behavior have not been established in the field of simulation training [14, 24, 25]. In addition, the problem of skills retention is one of the most important problems in the field of simulation training [4, 5, 16, 17]. Knowledge, skills, and confidence decline in a few months to 1 year after a simulation course with no interventions [4, 5, 9, 16, 19]. Several methods have been proposed to help participants retain knowledge and skills; for example, providing didactic or online lectures, and holding hands-on training sessions or simulation training courses regularly or several months after the course [15, 18–21]. However, the problem with these methods is that they are expensive in terms of time and effort. In this study, knowledge, skills, and confidence were maintained 4 months after completion of the simulation course. It also led to behavioral change, with a significant increase in the number of US examinations performed in daily practice. In this study, action records were conducted before and after the course. Recording one's actions is a common practice in cognitive behavioral therapy. By being aware of recording actions after they are completed, they can become more clearly aware of the purpose and content of actions, which leads to behavioral change. Therefore, it is possible that action record of this study had a positive impact on the increase in routine clinical ultrasound examinations after the course and the maintenance of skills 4 months later. This method involves less effort and cost than conventional methods and is feasible and can be implemented at most facilities. However, we didn't compare the two groups: one group that took the course only and the other group that took the course plus the action record, in this study. In order to examine the effect of the action record rigorously, it is necessary to compare the two groups. This is the subject of our next study.

This study had several limitations. First, this study involved a follow-up test 4 months after the course. Participants were aware of this follow-up test in advance, which might have influenced their behavior. However, all participants were informed that the results of this study would not affect their future work or training. Therefore, the impact of the follow-up test was not considered large. Second, skills were maintained 4 months after participating the course, but it is unclear to what extent the action record had a positive impact on skill maintenance because the course-only group was not directly compared to the course plus action record group. Third, skills decline within from a few months to a year after participating a simulation course without some intervention in general. Since this study was conducted after only 4 months, a longer-term follow-up study is needed.

## Conclusion

The POCUS simulation course for physicians is useful for JNPs to acquire US examination skills even if it is not arranged for JNPs. Image interpretation skills, image acquisition skills, and confidence improved significantly and were maintained four months after the course began. It leads to behavioral changes such as increasing the number of US examinations in daily practice after the course.

## Supplementary Information

**Additional file 1.** Image acquisition test check list Station “Focused Cardiac Ultrasound”.

**Additional file 2.** Confidence self-evaluation sheet.

**Additional file 3.** Microsoft Excel sheet for participants to record their ultrasound exam logs.

**Additional file 4.** Raw data of number of US examinations by category performed before and after the course.

**Additional file 5.** Actual number of US examinations performed after the course.

## Data Availability

The datasets used and analysed during the current study available from the corresponding author on request.

## Abbreviations

POCUS: Point-of-care ultrasound
US: Ultrasound
DVT: Deep vein thrombosis
JNP: Japanese nurse practitioner
SD: Standard deviation
