# Supplementary material for: Effectiveness of point of care ultrasound (POCUS) simulation course and skills retention for Japanese nurse practitioners
Source: BMC Nurs. 2023 Jan 23;22:21. doi: 10.1186/s12912-023-01183-2 (PMC9872333; doi:10.1186/s12912-023-01183-2)
Supplement: Supplementary file 1 — Additional file 1. Image acquisition test check list Station “Focused Cardiac Ultrasound”. [file 12912_2023_1183_MOESM1_ESM.docx]

**Image acquisition test check list**

**Station “Focused Cardiac Ultrasound”**

Participant’s ID　　　　　　　 Instructor’s name　　　 Date:

**Step1：Parasternal long axis and short axis view　 The number of “Yes”　　/11**

**Q: Acquire a parasternal long-axis view and point to the left atrium, left ventricle, mitral valve, aortic valve,**

**right ventricle, and epicardium.**

- Orientation marker on screen corrected to the right. （　Yes　・　No　）
- Probe is properly positioned （　Yes　・　No　）
- Gain is appropriate （　Yes　・　No　）
- Depth is appropriate （　Yes　・　No　）
- Appropriate view is acquired. （　Yes　・　No　）
- The six specified structures are identified （　Yes　・　No　）

**Q: Acquire the parasternal short-axis view at the level of papillary muscle, and point to the left**

**ventricle, septum, right ventricle, and papillary muscle.**

- Probe is properly positioned （　Yes　・　No　）
- Gain is appropriate （　Yes　・　No　）
- Depth is appropriate （　Yes　・　No　）
- Appropriate view is acquired. （　Yes　・　No　）
- The four specified structures are identified （　Yes　・　No　）

**Step2：Apical four-chamber view　 The number of “Yes”　　/5**

**Q: Acquire the apical four-chamber view and point to the left atrium, left ventricle, right atrium, right ventricle,**

**mitral valve, and tricuspid valve.**

- Probe is properly positioned （　Yes　・　No　）
- Gain is appropriate （　Yes　・　No　）
- Depth is appropriate （　Yes　・　No　）
- Appropriate view is acquired. （　Yes　・　No　）
- The six specified structures are identified （　Yes　・　No　）

**Step3：Subcostal four-chamber view and IVC view The number of “Yes”　　/10**

**Q: Acquire the Subcostal four-chamber view and point to the left atrium, left ventricle, right atrium, and right**

**ventricle.**

- Probe is properly positioned （　Yes　・　No　）
- Gain is appropriate （　Yes　・　No　）
- Depth is appropriate （　Yes　・　No　）
- Appropriate view is acquired. （　Yes　・　No　）
- The four specified structures are identified （　Yes　・　No　）

**Q: Acquire the Subcostal IVC view and point to the IVC, the inflow area to the right atrium, and the hepatic**

**venous inflow area.**

- Probe is properly positioned （　Yes　・　No　）
- Gain is appropriate （　Yes　・　No　）
- Depth is appropriate （　Yes　・　No　）
- Appropriate view is acquired. （　Yes　・　No　）
- The three specified structures are identified （　Yes　・　No　）

**Evaluation rubric of Focused Cardiac Ultrasound**

**Probe is properly positioned**

- **PLAX**：Supine or left lateral position. Probe position is between the 2nd-5th intercostal spaces along the left margin of the sternum. Probe marker pointing toward the right shoulder.
- **PSAX**：Supine or left lateral position. 90° clockwise from the PLAX view, with the probe marker pointing toward the left shoulder
- **Apical four-chamber view**: Supine or left lateral position. The probe is placed at the apex of the heart with the ultrasound beam pointing toward the right shoulder. Probe marker is on the patient's left-hand side.
- **Subcostal four-chamber view**: Supine position. The probe is placed at the cardiac fossa and the ultrasound beam pointing toward the heart. Probe marker is on patient's left-hand side
- **Subcostal IVC view**: Supine position. The probe is placed perpendicularly to the cardiac fossa. Probe marker is cephalad

**Gain is appropriate**

- All designated structures must be readily visible.

**Depth is appropriate**

- All structures to be observed should be well within the screen. They should not be missing or biased to the upper half of the screen.

**Criteria for Appropriate view: All three of the following are met for each view**

- **PLAX**

□Mitral valve is in the center of the screen.

□The right ventricle, aorta, and left atrium are observed to be approximately the same size

□The basal to mid-level of the left ventricle is observed (the apex is not observed).

- **PSAX (papillary muscle level)**

□Anterior and posterior papillary muscles are observed symmetrically

□Left ventricle observed as a regular circle

□Left ventricle is in the center of the screen.

- **Apical four-chamber view**

□Ventricular septum and atrial septum are observed in the center line of the screen

□Mitral and tricuspid valves are observed to open and close

□Both ventricles and atrium are observed at their largest visible point.

- **Subcostal four-chamber view**

□Mitral and tricuspid valves are observed to open and close

□Both ventricles and atria are observed at their largest visible point.

□The liver is an acoustic window

- **Subcostal IVC view**

□The confluence of the inferior vena cava and hepatic vein is observed.

□The right atrium is observed.

□Respiratory fluctuations in the IVC is observed

**Image acquisition test check list**

**Station “Lung Ultrasound and DVT US for the lower extremities”**

Participant’s ID　　　　　　　 Instructor’s name　　　 Date:

**Lung ultrasound: B mode, M mode　　 The number of “Yes”　　/8**

**Q: Indicate the four-evaluation point of lung ultrasound, and choose one point. Acquire the Lung sliding, A-line,**

**and Sea shore sign of the point.**

- He/ she can indicate all four-evaluation point of one lung. （　Yes　・　No　）
- Orientation marker on screen corrected to the right. （　Yes　・　No　）
- Probe is properly positioned （　Yes　・　No　）
- Gain is appropriate （　Yes　・　No　）
- Depth is appropriate （　Yes　・　No　）
- Appropriate view is acquired. （　Yes　・　No　）
- Lung sliding and A-line are identified （　Yes　・　No　）
- Sea shore sign is displayed. （　Yes　・　No　）

**Lung ultrasound: Diaphragm　　 The number of “Yes”　　/5**

**Q: Acquire diaphragm view in appropriate point.**

- Probe is properly positioned （　Yes　・　No　）
- Gain is appropriate （　Yes　・　No　）
- Depth is appropriate （　Yes　・　No　）
- Appropriate view is acquired. （　Yes　・　No　）
- Diaphragm is identified （　Yes　・　No　）

**DVT US for the lower extremities　 The number of “Yes”　　/7**

**Q: Indicate four evaluation point on the femoral region for lower extremity vascular echocardiography. Choose two of these points, perform the compression test, and indicate the artery and vein.**

- He/ she can indicate all four-evaluation point of femoral region. （　Yes　・　No　）
- Probe is properly positioned in both two points （　Yes　・　No　）
- Gain is appropriate （　Yes　・　No　）
- Depth is appropriate （　Yes　・　No　）
- Appropriate view is acquired in both two points. （　Yes　・　No　）
- Compression testing is done properly in both two points. （　Yes　・　No　）
- Arteries and veins are correctly identified. （　Yes　・　No　）

**Evaluation rubric of Lung Ultrasound**

**Probe is properly positioned**

- Lung ultrasound: Spin position. Probe marker is caudal. The following four locations,

1: Midclavicular line level of 2nd intercostal space

2: Level of anterior axillary line between the 5th ribs

3: Level of midaxillary line between 8th and 9th ribs

4: Level of posterior axillary line between 8th and 9th ribs

- Diaphragm：Spin position. Probe marker is caudal. Zone of apposition

**Gain is appropriate**

- All designated structures must be readily visible.

**Depth is appropriate**

- All structures to be observed should be well within the screen. They should not be missing or biased to the upper half of the screen.

**Criteria for Appropriate view**

- Lung sliding and A-line can be seen in intercostal spaces.
- Diaphragm: Liver and diaphragm can be observed. Lung is visible and hidden by breathing

**Evaluation rubric of DVT US for the lower extremities**

**Probe is properly positioned**

- DVT US for lower extremities: Spin position. Probe marker is on patient's left-hand side.

The following four locations,

1: Common femoral vein of inguinal area

2: Bifurcation of common femoral vein and great saphenous vein

3: Bifurcation of common femoral vein and lateral perforating branch

4: Bifurcation of shallow femoral vein and deep femoral vein

**Gain is appropriate**

- All designated structures must be readily visible.

**Depth is appropriate**

- All structures to be observed should be well within the screen. They should not be missing or biased to the upper half of the screen.

**Criteria for Appropriate view:**

See figure below: Right femoral region


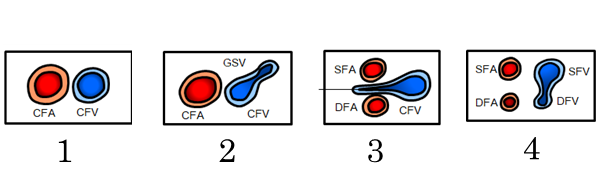


**Image acquisition test check list**

**Station “Abdominal Ultrasound”**

Participant’s ID　　　　　　　 Instructor’s name　　　 Date:

**Step1：Gall bladder The number of “Yes”　　/6**

**Q: Acquire a long-axis view of the gallbladder and perform the sonographic murphy test with verbal explanation.**

- Orientation marker on screen corrected to the right. （　Yes　・　No　）
- Probe is properly positioned （　Yes　・　No　）
- Gain is appropriate （　Yes　・　No　）
- Depth is appropriate （　Yes　・　No　）
- Appropriate view is acquired. （　Yes　・　No　）
- Appropriate description and technique of the Sonographic murphy test （　Yes　・　No　）

**Step2：Kidney・Abdominal aorta The number of “Yes”　　/10**

**Q: Acquire a long-axis view of the right kidney and point to the kidney and renal pelvis.**

- Probe is properly positioned （　Yes　・　No　）
- Gain is appropriate （　Yes　・　No　）
- Depth is appropriate （　Yes　・　No　）
- Appropriate view is acquired. （　Yes　・　No　）
- Kidney and renal pelvis are indicated （　Yes　・　No　）

**Q: Acquire the short and long axis view of the abdominal aorta.**

- Probe is properly positioned （　Yes　・　No　）
- Gain is appropriate （　Yes　・　No　）
- Depth is appropriate （　Yes　・　No　）
- Appropriate views are acquired for both short-axis and long-axis　 （　Yes　・　No　）
- Abdominal aorta is observed for both short-axis and long-axis （　Yes　・　No　）

**Step3：Bladder・Ascites　 The number of “Yes”　　/6**

**Q: Acquire the short and long axis view of the bladder. Indicate the three places to check for ascites.**

- Probe is properly positioned （　Yes　・　No　）
- Gain is appropriate （　Yes　・　No　）
- Depth is appropriate （　Yes　・　No　）
- Appropriate views are acquired for both short-axis and long-axis　 （　Yes　・　No　）
- Bladder is observed for both short-axis and long-axis （　Yes　・　No　）
- He/ she can indicate all three places to check for ascites. （　Yes　・　No　）

**Evaluation rubric of Abdominal Ultrasound**

**Probe is properly positioned**

- Gall bladder：Supine or left lateral position. Intercostal to below the costal arch at the right quadrant. Probe marker is not cephalad.
- Kidney：Spin position. Lateral abdomen between anterior and posterior axillary lines. Probe marker is caudal.
- Abdominal aorta: Spin position. Around the midline of the abdomen, between the cardiac fossa and the umbilicus. Probe marker is on patient's left-hand side in short axis view, and caudal in long axis view.
- Bladder: Spin position. Around the midline of the abdomen, around the superior border of the pubic symphysis. Probe marker is on patient's left-hand side in short axis view, and caudal in long axis view.

**Gain is appropriate**

- All designated structures must be readily visible.

**Depth is appropriate**

- All structures to be observed should be well within the screen. They should not be missing or biased to the upper half of the screen.

**Criteria for Appropriate view**

- Gall bladder: The maximum diameter of the long axis of the gallbladder is observed in the right quadrant.
- Kidney: The maximum diameter of the long axis of kidney, and the renal pelvis is observed.
- Abdominal aorta: The maximum diameter of the aorta is observed in the short and long axis, respectively.
- Bladder: The maximum diameter of the bladder is observed in the short and long axis, respectively.
