# Supplementary material for: Effectiveness of point of care ultrasound (POCUS) simulation course and skills retention for Japanese nurse practitioners
Source: BMC Nurs. 2023 Jan 23;22:21. doi: 10.1186/s12912-023-01183-2 (PMC9872333; doi:10.1186/s12912-023-01183-2)
Supplement: Supplementary file 2 — Additional file 2. Confidence self-evaluation sheet. [file 12912_2023_1183_MOESM2_ESM.docx]

**Adittional file 2: confidence self-evaluation sheet**

| Questions | Scoring |
| --- | --- |
| General skills and machine operations | 5-agree |
| 1. I am confident in general image acquisition skills | 4-mostly agree |
| 2. I am confident in general image interpretation skills | 3-neutral |
| 3. I am confident in machine operations | 2-mostly disagree |
|  | 1-disagree |
| FOCUS |  |
| 4. I am confident in evaluation skills for cardiac systolic function |  |
| 5. I am confident in evaluation skills for inferior vena cava |  |
|  |  |
| Vascular |  |
| 6. I am confident in evaluation skills for deep vein thrombosis |  |
|  |  |
| Lung/diaphragm |  |
| 7. I am confident in evaluation skills for pneumothorax |  |
| 8. I am confident in evaluation skills for pulmonary edema |  |
| 9. I am confident in evaluation skills for diaphragmatic function |  |
|  |  |
| Abdomen |  |
| 10. I am confident in evaluation skills for cholecystitis |  |
| 11. I am confident in evaluation skills for hydronephrosis |  |
| 12. I am confident in evaluation skills for aortic aneurysm |  |
| 13. I am confident in evaluation of ureteral catheter |  |
| 14. I am confident in evaluation skills for ascites |  |
|  |  |
| FOCUS: Focused cardiac ultrasound |  |

※This sheet is reference from BMC Med Educ. 2018;18(1):202.
