# Supplementary material for: Effectiveness of point of care ultrasound (POCUS) simulation course and skills retention for Japanese nurse practitioners
Source: BMC Nurs. 2023 Jan 23;22:21. doi: 10.1186/s12912-023-01183-2 (PMC9872333; doi:10.1186/s12912-023-01183-2)
Supplement: Supplementary file 3 — Additional file 3. Microsoft Excel sheet for participants to record their ultrasound exam logs. [file 12912_2023_1183_MOESM3_ESM.pdf]

Additional file 3: Microsoft Excel sheet for participants to record their ultrasound exam logs

Ultrasound exam log sheet after POCUS course

Examination date	Age	Sex	Location of US exam	Status of physician supervision	Domain	Purpose of US exam	examination findings
Describe the date the US exam was performed		M or F	Select the location where the ultrasound was performed from the following: "ward", "outpatient", "home visit"	A：Performed with a physician present next to you<br>B：Performed by yourself under the doctor's instruction and report to the doctor.(Physician was not present next to you)	Select from the following domein "FOCUS", "Lung", "DVT US of Lower extremities", and "abdomen"	Briefly describe the purpose of US exam	Briefly describe exam findings
