# Supplementary material for: Effectiveness of point of care ultrasound (POCUS) simulation course and skills retention for Japanese nurse practitioners
Source: BMC Nurs. 2023 Jan 23;22:21. doi: 10.1186/s12912-023-01183-2 (PMC9872333; doi:10.1186/s12912-023-01183-2)
Supplement: Supplementary file 4 — Additional file 4. Raw data of number of US examinations by category performed before and after the course. [file 12912_2023_1183_MOESM4_ESM.pdf]

Additional file 4: raw data of number of US examinations by category performed before and after the course

ID	Pre_course total	Pre_course CardiacUS	Pre_course LungUS	Pre_course DVT US	Pre_course Abdominal US	Post_course total	Post_course CardiacUS	Post_course LungUS	Post_course DVT US	Post_course Abdominal US
1	1	1	1	1	1	4	3	2	2	2
2	1	1	1	1	1	2	2	2	1	2
3	1	1	1	1	1	4	3	2	1	2
4	3	2	1	1	3	3	2	2	2	2
5	2	1	1	1	2	3	2	2	1	3
6	4	3	2	2	3	3	2	2	2	2
7	2	2	1	1	1	4	3	2	2	2
8	1	1	1	1	1	3	2	2	1	2
9	1	1	1	1	1	3	3	2	2	2
11	3	2	1	2	2	4	3	2	2	2
12	1	1	1	1	1	3	3	2	2	2
13	1	1	1	1	1	3	2	2	2	3
14	1	1	1	1	1	3	2	1	2	2
15	5	2	2	1	2	3	2	2	2	3
16	2	2	1	1	1	3	3	2	2	3
17	3	3	3	1	3	4	3	2	3	2
18	1	1	1	1	1	3	2	2	1	2
19	1	1	1	1	1	4	3	2	2	2
20	1	1	1	1	1	5	3	2	2	3
21	3	2	1	1	2	5	3	2	2	3
23	3	3	1	2	2	6	5	3	3	3
24	1	1	1	1	1	3	3	2	2	2
25	1	1	1	1	1	4	3	2	2	3
26	2	2	2	2	2	3	3	2	2	2
27	1	1	1	1	1	3	2	2	1	2
28	1	1	1	1	1	4	3	2	2	2
29	1	1	1	1	1	5	3	1	2	4
30	1	1	1	1	1	5	3	3	2	3
31	2	2	1	1	2	3	3	2	1	2
32	2	2	1	1	2	3	2	2	2	2
33	1	1	1	1	1	4	2	2	2	3
34	2	2	2	2	1	4	3	2	2	2
35	1	1	1	1	1	3	2	2	1	2

category 1: 0 cases, category 2: 1-9 cases, category 3: 10-29 cases, category 4: 30-49 cases, category 5: 50-99 cases, and category 6: 100 or more cases

※ID10 and 22 were excluded because they could not complete the course curriculum
