# Supplementary material for: Effectiveness of point of care ultrasound (POCUS) simulation course and skills retention for Japanese nurse practitioners
Source: BMC Nurs. 2023 Jan 23;22:21. doi: 10.1186/s12912-023-01183-2 (PMC9872333; doi:10.1186/s12912-023-01183-2)
Supplement: Supplementary file 5 — Additional file 5. Actual number of US examinations performed after the course. [file 12912_2023_1183_MOESM5_ESM.pdf]

Additional file 5: actual number of US examinations performed after the course

ID	Post_course total	Post_course CardiacUS	Post_course LungUS	Post_course DVT US	Post_course Abdominal US
1	40	21	7	4	8
2	9	1	5	0	3
3	32	14	9	0	8
4	14	8	2	3	1
5	20	7	3	0	10
6	17	9	1	1	5
7	32	24	4	1	3
8	14	7	2	0	5
9	20	13	2	2	3
11	35	20	3	3	9
12	16	11	1	1	3
13	23	8	3	2	10
14	20	6	0	6	8
15	17	2	1	2	12
16	27	10	2	1	14
17	45	19	4	15	7
18	25	5	5	0	9
19	30	15	2	4	9
20	61	24	9	2	25
21	50	28	6	6	10
23	110	71	16	11	12
24	27	15	5	4	3
25	38	10	4	4	20
26	29	17	5	4	3
27	15	9	1	0	5
28	34	16	7	4	7
29	60	23	0	3	34
30	60	24	10	5	21
31	26	20	1	0	4
32	19	7	4	2	6
33	31	8	5	4	14
34	34	22	5	3	4
35	14	7	2	0	5
mean	32	15	4	3	9

※ID10 and 22 were excluded because they could not complete the course curriculum
